# Classification of Breast Cancer Lesions in Ultrasound Images by Using Attention Layer and Loss Ensemble in Deep Convolutional Neural Networks

**DOI:** 10.3390/diagnostics11101859

**Published:** 2021-10-09

**Authors:** Elham Yousef Kalafi, Ata Jodeiri, Seyed Kamaledin Setarehdan, Ng Wei Lin, Kartini Rahmat, Nur Aishah Taib, Mogana Darshini Ganggayah, Sarinder Kaur Dhillon

**Affiliations:** 1 Data Science & Bioinformatics Laboratory, Institute of Biological Sciences, Faculty of Science, University of Malaya, Kuala Lumpur 50603, Malaysia; elham@um.edu.my (E.Y.K.); mogana@ummc.edu.my (M.D.G.); 2 School of Electrical and Computer Engineering, College of Engineering, University of Tehran, 1417935840 Tehran, Iran; ata.jodeiri@ut.ac.ir (A.J.); ksetareh@ut.ac.ir (S.K.S.); 3 Department of Biomedical Imaging, Faculty of Medicine, University of Malaya, Kuala Lumpur 50603, Malaysia; wei.lin@um.edu.my; 4 Department of Surgery, Faculty of Medicine, University of Malaya, Kuala Lumpur 50603, Malaysia; naisha@um.edu.my

**Keywords:** breast cancer, ultrasound, deep learning, diagnostic imaging, classification

## Abstract

The reliable classification of benign and malignant lesions in breast ultrasound images can provide an effective and relatively low-cost method for the early diagnosis of breast cancer. The accuracy of the diagnosis is, however, highly dependent on the quality of the ultrasound systems and the experience of the users (radiologists). The use of deep convolutional neural network approaches has provided solutions for the efficient analysis of breast ultrasound images. In this study, we propose a new framework for the classification of breast cancer lesions with an attention module in a modified VGG16 architecture. The adopted attention mechanism enhances the feature discrimination between the background and targeted lesions in ultrasound. We also propose a new ensembled loss function, which is a combination of binary cross-entropy and the logarithm of the hyperbolic cosine loss, to improve the model discrepancy between classified lesions and their labels. This combined loss function optimizes the network more quickly. The proposed model outperformed other modified VGG16 architectures, with an accuracy of 93%, and also, the results are competitive with those of other state-of-the-art frameworks for the classification of breast cancer lesions. Our experimental results show that the choice of loss function is highly important and plays a key role in breast lesion classification tasks. Additionally, by adding an attention block, we could improve the performance of the model.

## 1. Introduction

Breast cancer is the second leading cause of cancer death in women [1,2]. Automated techniques are proposed for building interoperability functions among different clinical departments such as the diagnosis, screening and treatment of breast cancer [3,4,5]. As parts of these automated systems, different types of imaging modalities such as mammography, ultrasound and magnetic resonance imaging have been used for diagnosing breast tumors. Whilst mammography has been proven to be a useful technique for diagnosing breast cancer leading to reduced mortality [6], its sensitivity is limited in dense breast tissues. Breast density has been established as an independent risk factor for breast cancer [7,8,9]. Women with heterogenous dense and extremely dense breast tissues have relatively higher risks, 1.2 and 2.1 times, of developing breast cancers compared to average women [10]. The accuracy rate for simple benign cyst diagnosis in breast ultrasound images has been reported to be 96–100%; therefore, they do not require further evaluation [11]. In a meta-analysis of 29 studies, various adjunct screening methods were studied to assess the limitations of various breast cancer screening modalities, and ultrasound has demonstrated an increase in cancer detection of 40% [12].

Computer-aided diagnosis (CAD) systems are extensively used for the detection and classification of tumors in breast ultrasound images. The CAD systems are highly suggested for helping radiologists in identifying breast tumors and disease prognosis. Statistical methods [13] have been predominantly used to analyze extracted features such as lesion shapes, margins, homogeneity and posterior acoustic attenuation. However, the identification of the shapes and margins of lesions is difficult in ultrasound images [14]. Machine learning techniques have also been extensively deployed to analyze and classify lesions based on the handcrafted morphological and texture features of tumors [15,16]. However, the extraction of features is still highly dependent on the radiologist’s experience. The struggles of researchers for handcrafting features led to the development of newer algorithms that can learn features automatically from data such as deep learning algorithms, which are particularly useful for extracting non-linear features from data. Deep learning models are surprisingly promising in the classification of ultrasound images, in which pattern recognition is not easily hand-engineered [17,18].

A large group of studies with deep learning approaches leverage the concept of pre-trained Convolutional Neural Networks (CNNs) to classify tumors in breast ultrasound images [19,20,21,22]. In [19], the pretrained GoogLeNet network was finetuned with their local dataset to differentiate benign and malignant tumors. In this strategy, the parameters of the modified pre-trained CNN model are fine-tuned on breast ultrasound images, and the last fully connected layer of the pre-trained CNN model is modified based on the number of classes in the classification problem. In [20], deep networks were applied to integrate feature learning with feature selection on breast shear wave elastography images. The stacked denoising autoencoder was used in [21] to differentiate lesions in breast ultrasound images. 

Along with the rapid growth of deep learning methods in the past few years, attention mechanisms are needed to efficiently integrate local and global features and exploit localized information [23]. The attention has been used in computer vision tasks such as detection [24,25], segmentation [26] and classification [27], and it improves the model performance by focusing on the most relevant features that are important in the given task. To the best of our knowledge, attention modules have been widely used in medical image segmentation but not classification. In this study, we used the attention gate module [23] in a modified VGG16 architecture with a new loss function to increase the classification performance of breast lesion classification in ultrasound images.

## 2. Materials and Methods

**Figure 1:**
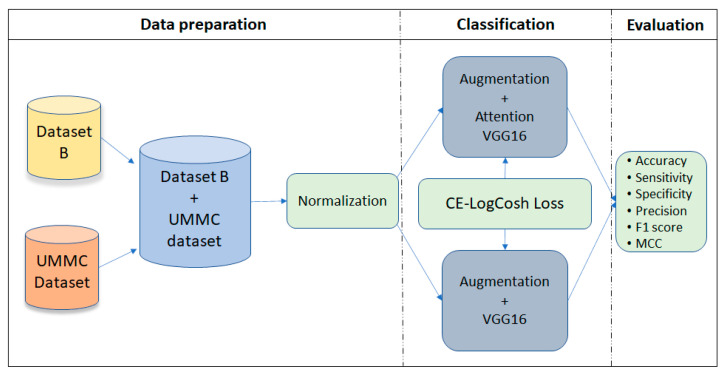
The overall framework of the proposed study. This splits into three phases: data preparation, lesion classification and evaluation. In data preparation, the two ultrasound image datasets (i.e., Dataset B and UMMC) were integrated and normalization. Classification step involves the proposed Attention-VGG16 model with CE-LogCosh loss function, which was then compared using the standard pre-trained VGG16 network. Finally, the classification performance was measured according to the six evaluation metrics.

The proposed framework in this study was inspired by [23], in which the authors introduced attention gates. We used a layer with attention mechanism in the modified VGG16 architecture. The attention block was used in convolutional layers 13 and 18 in the VGG16 network architecture to extract and prune features [28]. The framework is illustrated in Figure 1.

### 2.1. The Datasets

In this study, we combined two datasets, one publicly available set of breast ultrasound images, named Breast Ultrasound Database B [29], with 163 images of 109 benign and 54 malignant lesions, and another dataset that was collected at the University Malaya Medical Centre (UMMC), between June 2012 and April 2013, with 276 ultrasound breast images comprising 140 benign and 136 malignant lesions obtained from 83 different patients. All the subjects were biopsy confirmed. The patients were either from the breast assessment clinic with palpable lumps or had sonographically detected lesions. Patients without confirmed histological diagnoses and those with a previously known histological diagnosis were excluded.

Most of the malignant lesions were infiltrating ductal carcinomas (IDCs), whereas the majority of the benign lesions were fibroadenomas. The sizes of the malignant lesions ranged from 0.5 to 9.0 cm (mean ± SD: 2.1 ± 1.2 cm), whereas the sizes of the benign lesions ranged from 0.3 to 5.0 cm (mean ± SD: 1.4 ± 1.0 cm).

### 2.2. Data Pre-Processing

**Figure 2:**
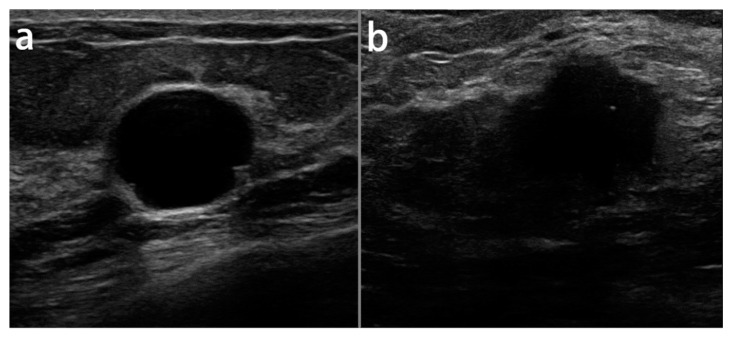
The samples of benign (**a**) and malignant (**b**) lesions in breast ultrasound images.

All the ultrasound images in the UMMC dataset were acquired using the Aixplorer ultrasound system (SuperSonic Imagine, Aix en Provence, France) using a 15-4 MHz linear transducer probe. Two radiologists specialized in breast imaging performed the scanning task, and they were blinded to the histological diagnosis results. All the images were in JPEG format and at the resolution of 1400 × 1050 pixels. However, the average image resolution size in dataset B was 760 × 570 pixels, where each of the images presented one or more lesions. In our experiments, the images were resized to 128 × 128 pixels and the whole data were split 75%, 10% and 15% for training, validation and test sets, respectively. Image normalization was applied to all of the images in the datasets to create a consistent dynamic range across the dataset. Figure 2 illustrates the samples of benign and malignant lesions in the breast ultrasound images of the UMMC dataset.

### 2.3. Attention Module

**Figure 3:**
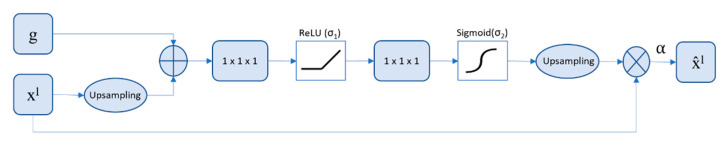
Illustration of attention gate (AG). Feature map upsampling was computed by bilinear interpolation.

The main idea of the attention mechanism is to highlight relevant features and suppress irrelevant components such as shadows or speckle noises in US images from background regions, with no ROI cropping required. At the deep levels of convolutional layers, the network acquires the richest possible feature representation. Early CNN layers capture low-level features (i.e, shapes, edges, texture and intensity), whereas deep layers extract higher-level features [30,31]. Here, in this study, we used the attention coefficient to give scaler attention to targeted lesion regions. We used the attention gate (AG) [23] mechanism to highlight the relevant feature representations to discriminate between the lesion and non-lesion pixels in US images. This allows ignoring the artifacts from US images which significantly improves the results. Through the AG, the input feature map was submitted to element-wise multiplication with the attention coefficient to highlight the salient features; see Figure 3.

The attention gate parameters (Ꞝ_attn_) contain a set of linear transformations $W_{x}^{T}$, $W_{g}^{T}$ and б^T^ and bias terms b_g_ and bб. The attention coefficients δ_i_ ∈ [0, 1] are produced by AG at each pixel *i* to scale the input features $x_{i}^{l}$ and output features x^il . The localization of focus regions is obtained using the gating signal, g, for each pixel *i*. The gating signal is retrieved from a coarser scale than the input features $x_{i}^{l}$. The linear attention coefficients are computed using the element-wise sum of the *W*_x_, b_б_, *W*_g_ and b_g_ parameters followed by 1 × 1 × 1 linear transformation б^T^ [23]:

(1) $$A_{attn}^{l}=\;{б}^{T}\;(\sigma_{1}\;(W_{x}^{T}x_{i}^{l}+W_{g}^{T}g_{i}+b_{g}))+b{б}_{\;},$$

(2) $$\delta_{i}^{l}=\sigma_{2}\;(A_{attn}^{l}\;(x_{i}^{l},\;g_{i};\;{Ꞝ}_{\mathrm{attn}})),$$

ReLU and sigmoid as σ_1_ and σ_2_, respectively, were used to transform the intermediate maps in calculating the attention coefficients. The attention coefficients determine the important regions of image and prune features to maintain the relevant activations in specific tasks. The output maps at each scale were upsampled and then concatenated with the pruned features. In this stage, 1 × 1 × 1 convolutions and non-linear activations were applied on each output map, and then, the high-dimensional feature representation was supervised with CE-logCosh loss.

### 2.4. Cross Entropy—Log Hyperbolic Cosine (CE-LogCosh) Loss

According to importance of the loss function in the learning algorithms, this study was inspired by the ensembled methods [32] in order to develop an ensemble loss function for the classification of breast ultrasound images. We combined two loss functions, cross entropy [33] and log hyperbolic cosine [34], to boost the learning process and achieve better performance. The cross-entropy loss compares the distribution of predictions and true labels and defines it as:

(3) LCE (y,y^)=−∑iyi logy^i,

The log-cosh loss function is the hyperbolic cosine algorithm of the prediction error.

(4) LLCH (y,y^)=∑i logcoshy^i−yi,

where y is the label and ${\hat{y}}_{i}$ is the predicted label. The proposed ensembled loss function is formulated as follows:


(5) $$\mathrm{CE-logCosh}\;\mathrm{Loss}=\alpha \;L_{\mathrm{CE}}+\beta \;L_{\mathrm{CCH}},$$

In the CE-logCosh Loss function, α and β are weighting parameters that can be tuned to shift the emphasis on the cross entropy or logCosh loss. In this study, we set α and β to 0.5, as the best performance was achieved.

### 2.5. Network Architecture

**Figure 4:**
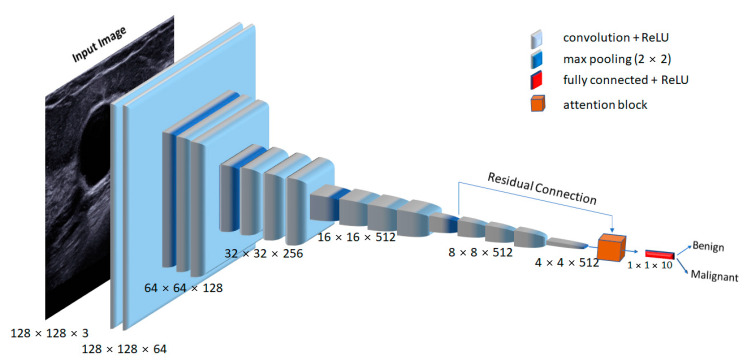
Proposed attention-VGG16 network. The extracted features from convolutional layer 13 were input to the attention block through a residual connection. The other input to the attention block was the feature maps from the 18th convolutional layer.

In this study, we used a trained VGG16 network to extract relevant features from the ultrasound datasets. Figure 4 presents the schematic of the proposed network architecture. The input image size for the VGG16 network was 128 × 128 × 3. Each convolutional layer used a kernel size of 3 × 3, followed by a non-linear ReLU activation function. After every convolutional block, a max-pooling operation with a stride of 2 was used to downsample the extracted feature maps. We applied the attention mechanism to convolutional layers 13 and 18 to boost the feature discriminability, which captured the most relevant features by ignoring the irrelevant ones. Finally, the enhanced feature output was fed to modified fully connected (FC) [35] layers for the classification of malignant and benign lesions.

Due to the limitation of the small breast ultrasound image dataset, training a deep network from scratch was not feasible and would have caused overfitting. Therefore, to overcome this issue, we replaced the fully connected layer 1000 classes from the ImageNet pre-trained network to two classes for the classification into benign and malignant in breast ultrasound images. The “dropout” strategy [36] was also used to avoid overfitting. The experiment was trained for 250 epochs with a batch size of 32. The model was optimized using RMSprop with an initial learning rate of 2 × 10^−6^, which decayed by 10^−6^. All the experiments were performed using the deep neural networks in the Keras framework with the TensorFlow backend. 

We propose a new model based on attention gating and new loss function to enhance the performance of classification for breast ultrasound images. 

### 2.6. Evaluation

In this study, we measured the classification performance of models by using six evaluation metrices: the sensitivity, specificity, accuracy, precision, F1 score and Matthews correlation coefficient [37], which were obtained from confusion matrix entries. In a confusion matrix, the relation between classification outcomes and predicted classes is illustrated. The level of classification performance was calculated from the number of correct and incorrect classified samples in each class. The accuracy was computed based on the total number of correct predictions, defined as:

(6) $$\frac{TP+TN}{TP+FN+TN+FP}$$

The sensitivity is the proportion of true positives that are identified correctly, defined as:

(7) $$\frac{TP}{TP+{FN}^{\prime }}\;$$

The specificity is the proportion of true negatives that are correctly predicted, defined as:

(8) $$\frac{TN}{TN+{FP}^{\prime }}$$

The precision or positive predictive value is the ratio of correctly predicted positive observations to the total predicted positive observations, defined as:

(9) $$\frac{TP}{TP+FP}$$

The F1 score is the weighted average of the precision, which is calculated as:

(10) $$\frac{2TP}{2TP+FP+FN}$$

The Matthews correlation coefficient (MCC) is the coefficient of the correlation between the observed and predicted classifications, defined as:

(11) $$\frac{TP*TN-FP*FN}{\sqrt{\left(TP+FP\right)*\left(TP+FN\right)*\left(TN+FP\right)*{\left(TN+FP\right)}^{\prime }}},$$

where true positive (*TP*) and true negative (*TN*) refer to the numbers of correct predictions and false positive (*FP*) and false negative (*FN*), those of incorrect predictions.

## 3. Results

We evaluated our proposed model for the classification of ultrasound breast lesions into benign and malignant. In particular, the accurate classification of benign and malignant lesions in ultrasound images is a challenging task in the presence of various artifacts such as variety in shape, a low signal-to-noise ratio, ill-defined boundaries and poor contrast. We compared the proposed Attention-VGG16 model with the standard VGG16 network by utilizing a combination of different loss functions (i.e., pls add all the losses ).

**Figure 5:**
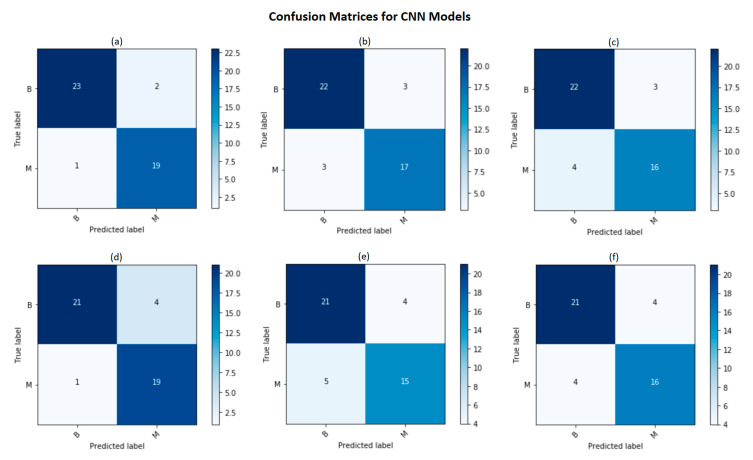
Illustration of confusion matrices for different models. (**a**) Attention-VGG16 with CE-logCosh loss, (**b**) Attention-VGG16 with CE loss, (**c**) Attention-VGG16 with logCosh loss, (**d**) VGG16 with CE-logCosh loss, (**e**) VGG16 with CE loss, and (**f**) VGG16 with logCosh loss.

According to Table 1 and Figure 5, it is notable that our proposed Attention-VGG16 model with CE-logCosh outperformed other classification models in terms of the accuracy, sensitivity, specificity, precision, F1 score and MCC.

## 4. Discussion

**Figure 6:**
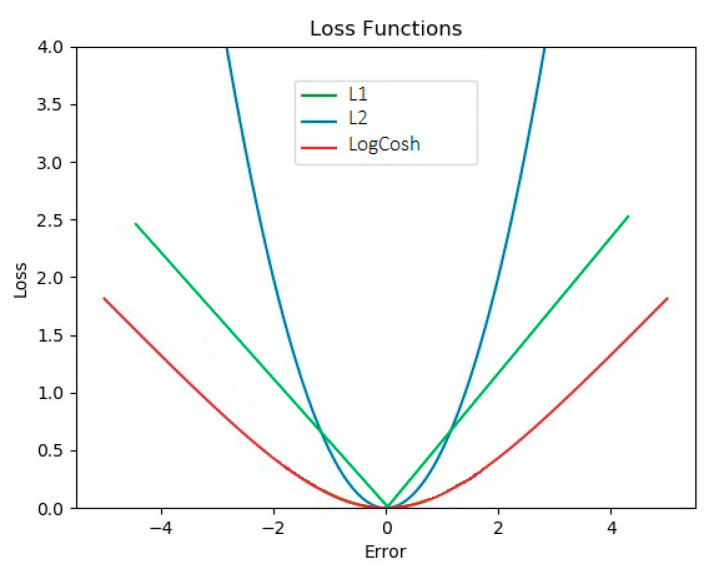
The comparison of L1, L2 and logCosh losses.

In this paper, modified VGG16 architectures were compared to achieve higher performance in the classification of benign and malignant breast tumors. Modifications such as an additional attention block, different dense layers and ensembled loss functions were made. One of the improvements in the CNN models was the use of ensembled loss functions. Within the training phase, in the gradient propagation optimization, the weight of each loss function was tuned, and they were parametrized by α and β to control the emphasis. To the best of our knowledge and according to Equation (4), logCosh loss works mostly like L2 at small values and like L1 at large values and is usually used in regression or reconstruction tasks [34]. In Figure 6, it is notable that the logCosh loss could obtain the benefit of L2 loss’ smoothness and the sharpness of L1.

In this study, we used logCosh loss, combined with binary cross-entropy, to improve the classification accuracy. It is notable in Table 1 that the ensemble of both losses could improve the classification performance.

On the other hand, by using an attention block, relevant spatial information was identified from low-level feature maps and propagated to the classification stage. The lack of this relevant spatial information is caused by transforming large sized feature maps that are obtained after the convolutional layers in the CNN and reaching smaller feature dimensions. Therefore, the attention block was proposed, which attempts to compute the contribution of each feature.

In our study, out of all the models, the Attention-VGG16 with logCosh loss demonstrated the highest accuracy and precision. Additionally, the proposed deep convolutional neural network architecture does not need prior expert knowledge or image segmentation; hence, it will be more convenient in CAD and suitable for future clinical diagnosis.

Table 2 demonstrates some state-of-the-art deep learning models for lesion classification for breast ultrasound images. It is notable that the performance of our proposed model is comparable to these published models [19,21,22].

One of the hyperparameters that was assessed in this study was the number of neurons in the dense layers. We used the smallest number of neurons to decrease the number of parameters, and surprisingly, this achieved the same accuracy as using 4096 or 10 neurons in dense layers.

In summary, we propose the Attention-VGG16 classifier as a potential architecture for classifying breast cancer ultrasound images. We suggest that this model be tested further using a larger dataset to improve the robustness of this architecture. Additionally, we also suggest that the VGG16 is implemented with machine learning classifiers as potential architectures in clinical studies. As the classification of breast lesions’ subtypes is of greater clinical impact [16,38], in future studies, the deep convolutional neural network architecture should be used on big data with various tumor subtypes to adapt it to multi-class classification.

## 5. Conclusions

In this study, we analyzed CNN models for the classification of benign and malignant lesions for the UMMC breast ultrasound image dataset and Breast Ultrasound Dataset B. We employed transfer learning approaches with the pre-trained VGG16 architecture. Different CNN models for classification were trained to predict benign or malignant lesions in breast ultrasound images. Our experimental results demonstrated that the choice of loss function is highly important in classification tasks and that adding an attention block could improve the performance of the proposed model. Additionally, the proposed model with extracted features from VGG16 and a fully connected network with only 10 neurons achieved the best performance in the classification task with respect to the precision of 92% and accuracy of 93%. With this framework, evaluation tests show that the combination of loss functions can provide suitable information to enable the construction of the most accurate prediction model when compared with other models. In the future, other deep neural network models will be tested on a larger dataset of ultrasound images with the hope of further increasing the accuracy and performance.

## Figures and Tables

**Table 1:** The comparison of the vgg16 and attention-vgg16 models with different loss functions in classification of benign and malignant lesions.

Models	Loss	Sensitivity	Specificity	Precision	Accuracy	F1 Score	MCC
VGG16	CE	0.80	0.78	0.84	0.80	0.82	0.59
VGG16	LogCosh	0.84	0.80	0.84	0.82	0.84	0.62
VGG16	CE-logCosh	0.95	0.82	0.84	0.89	0.89	0.79
Attention- VGG16	CE	0.88	0.85	0.88	0.87	0.88	0.73
Attention- VGG16	LogCosh	0.85	0.84	0.88	0.84	0.86	0.68
Attention- VGG16	CE-logCosh	0.96	0.90	0.92	0.93	0.94	0.87

**Table 2:** The state of the art of deep learning models for breast ultrasound lesion classification.

References	Dataset	Deep Learning Models	Performance
[19]	4254 benign	GoogLeNet	Accuracy: 91.23%
	3154 malignant		Sensitivity: 84.29%
			Specificity: 96.07%
[21]	275 benign	Stacked denoising Autoencoder	Accuracy: 82.4%
	245 malignant		Sensitivity: 78.7%
			Specificity: 85.7%
[22]	100 benign	Deep Polynomial network+SVM	Accuracy: 92.40%
	100 malignant		Sensitivity: 92.67%
			Specificity: 91.36%
Current Study	249 benign	Attention-VGG16 + ensembled loss	Accuracy: 93%
	190 malignant		Sensitivity: 96%
			Specificity: 90%

## Data Availability

The dataset by University Malaya Medical Center is protected and proprietary to internal staff. The public “Breast Ultrasound Dataset B” is available at http://www2.docm.mmu.ac.uk/STAFF/M.Yap/dataset.php (accessed on 29 September 2021).
